# A comprehensive review of the phytochemicals, health benefits, pharmacological safety and medicinal prospects of *Moringaoleifera*

**DOI:** 10.1016/j.heliyon.2024.e27807

**Published:** 2024-03-08

**Authors:** Emma Camilleri, Renald Blundell

**Affiliations:** a Department of Physiology and Biochemistry, Faculty of Medicine and Surgery, University of Malta, Imsida, MSD2080, Malta; b Centre for Molecular Medicine and Biobanking, University of Malta, MSD2080, Msida, Malta

**Keywords:** *Moringa oleifera*, Phytochemicals, Health benefits, Pharmacological potential, COVID-19

## Abstract

*Moringa oleifera* has emerged as a subject of increasing interest, drawing attention for its diverse phytochemical composition and potential health benefits. This review delves into *Moringa oleifera*'s phytochemical constituents, including but not limited to flavonoids, alkaloids, and carotenoids. Expanding beyond its chemical intricacies, the spectrum of health advantages attributed to it are explored, encompassing its remarkable anticancer, antioxidant, anti-diabetic, anti-inflammatory, antimicrobial, and cardioprotective effect. Throughout this review, the underlying physiological mechanisms attributed to these properties by its phytochemicals are explored. Concurrently, the review addresses its pharmacological safety, ensuring a nuanced understanding of its applications in medicinal industries. In summary, this literature review presents a comprehensive exploration of *Moringa oleifera*, focusing on its phytochemical composition, health benefits, physiological mechanisms, pharmacological safety and nutritional importance.

## 1 Introduction

*Moringa oleifera*, a plant native to South Asia known by various names such as the ‘drumstick tree’ or ‘horseradish tree,’ has drawn global attention due to its nutritional and therapeutic properties. Traditional medicine systems across cultures have long recognised its range of health benefits and pharmacological potential [1,2]. As the focus on natural remedies for contemporary health challenges grows, *Moringa oleifera* (MO) has become the subject of extensive research into its phytochemical composition, mechanisms of action, and health advantages.

This review explores the nutrients and phytochemicals in MO, which are the bioactive compounds responsible for its medicinal properties. These include but are not limited to flavonoids, alkaloids and carotenoids. Throughout, the physiological mechanisms and diverse pharmacotherapeutic properties like anticancer, antimicrobial, antioxidant and anti-inflammatory effects are examined [3]. Additionally, the safety of MO consumption, particularly its potential interactions with cytochrome enzymes is considered [4]. The environmental factors affecting MO's composition and overall impact on health are also acknowledged.

Therefore, the aim of this comprehensive literature review is to provide an updated and thorough examination of MO, addressing its phytochemical constituents, elucidating its diverse health benefits, and unravelling its physiological mechanisms as well as pharmacological safety. The scope of this review encompasses not only MO's established roles in traditional medicine but also its emerging importance in modern medicinal industries. Through an analysis of recent scientific literature, this review seeks to highlight MO's potential contributions to addressing various health concerns, ranging from chronic diseases to nutritional deficiencies.

## 2 Methodology

### 2.1 Literature search strategy

To comprehensively explore the diverse facets of *Moringa oleifera*, a literature search was conducted across various electronic databases, including PubMed, Scopus, and Google Scholar. The search was carried out using relevant keywords and combinations, such as “*Moringa oleifera*,” “phytochemicals of *Moringa oleifera*,” “health benefits of *Moringa oleifera*,” “anticancer properties of *Moringa oleifera*,” “antioxidant properties of *Moringa oleifera*,” “anti-diabetic properties of *Moringa oleifera*,” “anti-inflammatory properties of *Moringa oleifera*,” “antimicrobial properties of *Moringa oleifera*,” “COVID-19 and *Moringa oleifera”* and “cardioprotective effects of *Moringa oleifera*."

### 2.2 Inclusion and exclusion criteria

Inclusion criteria included studies detailing the phytochemistry, health benefits and physiological mechanisms underlying the medicinal attributes of MO together with studies highlighting the pharmacological safety and toxic effects of MO. Animal and human studies depicting such properties of MO were included too.

Exclusion criteria comprised of any articles going into the phytochemistry and health benefits of other Moringa species, industrial applications of MO, duplicated studies and studies going into exhaustive molecular detail on the plant's phytochemicals mechanisms. Articles that were not written in English, contained irrelevant or insubstantial data and articles where the full text was inaccessible were excluded from this review. Additionally, studies not directly related to MO and ones lacking substantial information on health benefits or mechanisms of action were also excluded.

## 3 The *Moringa oleifera* tree and its composition

### 3.1 Morphology and cultivation

MO grows in a versatile of conditions and is packed with a myriad of nutrients and bioactive compounds that are beneficial to both the medicinal and socioeconomic industries [3]. This magnoliophyte which is one of thirteen species belongs to the Morignaceae family and is native to the Northern Indian Himalayan Mountains [1,5]. MO has become naturalised in several countries, including Southwest Asia, Madagascar, Sir Lanka, Pakistan, Afghanistan, Bangladesh and Southwest and Northwest Africa [6,7].

MO thrives best under direct sunlight at temperatures ranging between 25 °C and 35 °C in marginally acidic or neutral soil (pH 4.5–9) at altitudes of about 2,000 m. However, it can withstand both hotter and colder climates, various soil conditions and even dry seasons. Being an adaptable deciduous tree enables it to grow expeditiously over a short period reaching a height of around 10 m [[8], [9], [10]].

MO has a supple trunk, gummy bark, green pinnate leaves and numerous white odoriferous zygomatic flowers. The seeds it produces are dispersed by wind and will remain viable for up to one year. Whilst MO can grow fruit during its first six to eight months, it is noted that limited amounts of fruit are yielded in its first two years [9]. Nevertheless, its production increases later on in life. Furthermore, MO has tuberous, stocky roots and can live for about twenty years [6].

### 3.2 The nutrients in *Moringa oleifera* and its vitality in malnourishment

Although each part of the tree – the leaves, pods, flowers, roots and bark – are consumable, studies have proven that the alkaloid and spirochin components in the roots which is a common alternative for horseradish are quite toxic and are consequently avoided. Furthermore, the tree's leaves are recognised as the safest edible part and are thus the most consumed [11]. Although MO is rich in minerals and vitamins, the majority of the calcium content in its leaves, is in the form of calcium oxalate crystals which unfortunately cannot be utilised by the body [10]. Nevertheless, unlike most vegetables which lose some of their nutrient content when cooked, MO leaves whether cooked, ground or not are an exception to this. In fact, the iron content triples when the leaves are boiled or pulverised into a fine powder [3]. In saying this, although the components of MO are known for their rich nutrient content its composition is not constant but is dependent on several factors amongst them the surrounding environment including soil composition, the weather, the type of fertilisers administered if any, the management of the crop pre- and post-harvesting, the age of the tree and how the plant is taken care of unless it is wild [8,10].

Additionally, MO leaves have seventeen types of fatty acids and all the essential amino acids. Together with this knowledge and the high content of inorganic minerals the ash provides, MO can be of benefit for vegans and vegetarians and can help prevent malnourishment if incorporated into the diet [11,12]. In fact, in poor countries, when MO was amalgamated into the diet, people's health was enhanced, a decrease in pregnant women having anaemia was observed, pregnant women delivered higher weight babies, breast-feeding mothers experienced an increase in lactation due to MO's galactagogic effect and infants achieved a healthier weight [12]. Furthermore, pregnant women are advised to take MO due to its particular high folate and omega-3 and omega-6 content which can help prevent birth defects, especially those concerning the nervous system. In addition, MO is a great plant to grow in such countries since the cultivation process is inexpensive, it grows all year round and they thrive in dry conditions when most vegetables and fruits are normally scarce during dry periods. It also does not require much attention or any specific conditions to grow [12]. Furthermore, one must note that although MO can be used to remedy mild to moderate malnourishment, severe malnourishment cannot be medicated using MO [12].

### 3.3 The phytochemicals in *Moringa oleifera*

Studies have found around 110 bioactive chemicals in MO which each have a protective or therapeutic attribute. The phytonutrient composition in each part of the tree varies as observed in Table 1. Apart from being a highly nutritious and multipurpose tree, MO is loaded with a myriad of phytonutrients that help protect against diseases as well as treat them. Furthermore, the composition of the phytochemistry in the plant varies depending on the part of the tree and the abiotic factors of the ecosystem it is grown in Ref. [6]. The phytochemicals found in MO can be grouped into six main groups: flavonoids, phenolic acids, glucosinolates, terpenes, alkaloids and sterols [3,13].

**Table 1:** *The phytochemistry of different parts of the plant in the* MO ***tree***^3,6^.

Part of MO	Phytochemicals Present	The Medicinal Properties of these Phytochemicals
Leaf	n-hexadecanoic acid, tetradecanoic acid, *cis*-vaccenic acid, octadecanoic acid, palmitoyl chloride, beta-*l*-rhamnofuranoside, 5-*O*-acetyl-thio-octyl, gamma-sitosterol, and pregna-7-diene-3-ol-20-one.	Anti-inflammatory, antioxidant, antidiabetic, antihyperlipidemic, antihypertensive, hepatoprotective, anticancer, antimicrobial
Radicle	4-(α-L-rhamnopyranosyloxy)- benzylglucosinolate and benzylglucosinolate.	Anti-bacterial, anti-fungal, anticancer.
Roots	Spirochin and anthonine.	Anti-inflammatory, analgesic, antipyretic, antimicrobial.
Peduncle	Beta-sitosterone, vanillin, 4-hydroxymellein, β-sitosterol, and octacosanoic acid.	Anti-inflammatory, antioxidant, hypocholesterolemic, antidiabetic, anticancer.
Stem	Moringine, moringinine, 4-hydroxymellein, octacosanoic acid, and β-sitosterol.	Anti-inflammatory, antioxidant, antidiabetic, anticancer.
Gum	Leucodelphinidin-3-*O*-B-d-galactopuranosy (1- >4)-*O*-B-d-glucopyranoside, l-arabinose, d-mannose, d-xylose, and d-galactose, l-rhamnose and d-glucuronic acid.	Prebiotic, antioxidant, immunomodulatory, anti-cancer.
Flowers	Rhamnetin, isoquercitrin, and kaempferitrin.	Antioxidant, anti-inflammatory, anticancer.
Pods	Isothiocyanate, thiocarbamates, nitrile, O-[2-hydroxy-3-(2-heptenyloxy)]-propyl undecanoate, methyl-*p*-hydroxybenzoate, and O-ethyl-4-[(α-*l*-rhamnosyloxy)-benzyl] carbamate.	Antimicrobial, anticancer, antidiabetic.
Seeds	benzylglucosinolate, 4-(α-*l*-rhamnopyranosyloxy)-benzylglucosinolate, 4-(α-*l*-rhamnosyloxy) benzylisothiocyanate, 4-(α-lrhamnosyloxy) phenylacetonitrile, and O-ethyl-4-(α-lrhamnosyloxy) benzyl carbamate.	Antimicrobial, anticancer, antidiabetic.

Phenolic components in MO mainly constitute of phenolics, flavonoids and phenolic acids. Although, such phytochemicals are distributed throughout the plant, the seeds are particularly rich in phenolic acids and flavonoids, especially the polyphenols gallic acid, ellagic acid and quercetin amongst others [14]. Flavonoids are commonly present in their flavanol and glycoside forms. The flavonoids myricetin, rhamnetin and kaempferol are some of the most ambulant flavonoids observed in MO out of all 26 flavonoids. In addition, eleven phenolic acids are seen in the leaves of the genus amongst them caffeic, chlorogenic, ferulic and syringic acids. Both flavonoids and phenolic acids have been proven to have antioxidant, anti-photoaging and anti-cancerous properties. However, its rich phenolic acid content is the main driving force for MO's effective scavenging activity that contributes significantly to its overall antioxidant, anti-inflammatory and hepatoprotective properties [10,15].

A pyronase moiety that is anomerically attached to O-sulfated (Z)-thiohydroximate produces a glucosinolate. Glucomoringin (4-*O*-(-L-rhamnopyranosyloxy)-benzyl glucosinolate) is the commonest glucosinolate in MO with moringin being the respective isothiocyanate. Isothiocyanates are produced from glucosinolates when plant tissue is disturbed which results in myrosinase liberation that will attach itself to a glucosinolate forming an isothiocyanate [15]. Anti-inflammatory and anti-bacterial actions are exhibited by these components via the stimulation of detoxification enzymes. Isothiocyanates demonstrate apoptotic properties too which serve as an anti-cancerous characteristic. Furthermore, both glucosinolates and isothiocyanates protect against hyperglycaemia [13].

Carotenoids are the major tetraterpenoid group found in the leaves of MO with the compound, lutein, being the main carotenoid. E-luteoxanthin, 15-Z-β-carotene, lupeol acetate, β-amyrin, α-amyrin, 13-Z-lutein and all-E-zeaxanthin carotenoids have also been isolated in MO. Tetraterpenoids' antioxidant properties play a protective role against numerous diseases by trapping free radicals that can result in cell architecture disruption and inflammation [10,15]. Furthermore, these phytoconstituents attribute to MO's natural broad spectrum antimicrobial properties against predominant periodontal pathogens. This makes it a suitable agent for oral health in preventing periodontal disease progression [2].

Some identified alkaloids in MO are marumoside A, marumoside B, pyrrolemarumine-4-*O*-α-l-rhamnopyranoside. Alkaloids, especially, N,α-l-rhamnopyranosyl exhibit a cardioprotective role against hypertension [13]. Additionally, the two main sterols extracted from *Moringa oleifera*'s bark and leaves were β-sitosterol-2-*O*- β-d-galactopyranoside and β-sitosterol respectively which depicts an anti-inflammatory role by inhibiting the secretion of inflammatory factors [13,15]. In addition, β-sitosterol can decrease the absorption of dietary cholesterol by the intestines [12].

## 4 The pharmacotherapeutic properties of *Moringa oleifera*

Whilst medicinal drugs aid one's immune system to recover from or manage an illness, they do have their limitations such as adverse drug reactions, drug-drug interactions, patient compliance, availability and affordability, especially if treating a chronic disease. Thus, the thirst for new alternatives that offer maximal benefits with minimal harm has shed light on traditional and herbal medicine [16,17]. Luckily, MO can act as an alternative natural remedy since its rich phytochemistry and bioactive chemical content allows it to target many diseases like hyperglycaemia, hypertension, migraine, cancer, infectious diseases and more [11].

### *4.1* *Moringa oleifera* and cancer

#### *4.1.1* *Moringa oleifera*'s phytoconstituents and properties against neoplasms

Extracts obtained from different parts of the plant are rich in the following phytochemicals but not limited to β-sitosterol-3-*O*- β-d-glucopyranoside, benzyl isothiocyanate, carbomates, quercetin, moringin, kaempferol, palmitic acid and α-tocopherol [6,12]. These bioactive chemicals found in MO contribute to several anti-cancer properties. First, MO shields healthy cells from oxidative DNA damage caused by cancerous chemicals [12]. It also plays a role in reducing the proliferation, angiogenesis, and metastases of tumours through its ability to induce apoptosis and its cytotoxic effects on neoplastic cells [6,18]. MO further prevents genetic mutations that can lead to cancer development [19]. Additionally, it promotes the restoration of hepatic cytochrome *b*5, cytochrome P450, and glutathione-S-transferase (GST) levels to normal [3]. Moreover, the hydroalcoholic drumstick extract of MO encourages cell cycle arrest and increases enzyme activity in Phase-I and Phase-II [3,20]. These combined effects highlight the potential of MO in combating cancer and its various mechanisms of action.

#### *4.1.2* *Moringa oleifera* and chemotherapy

Whilst chemotherapy is easily the gold standard treatment for advanced neoplasms, the adverse effects and multi-drug resistance seen in neoplasms have brought their challenges in the battle against cancer [21]. Fortunately, tumours exhibit little resistance to MO and its extracts show minimal unwanted reactions and low toxicity, unlike many chemotherapeutic agents [21]. Studies have also proven that the antioxidant and free radical scavenging attributes of saponins found in MO extracts protect non-cancerous cells from polycyclic aromatic hydrocarbons (PAH7) and 12-dimethylbenz anthracene (DMBA) damage connoted by chemotherapy [3,18]. Additionally, combining doxorubicin with MO extracts displays a remarkable synergistic effect in HeLa cells where their proliferation is significantly reduced, highlighting further MO's potential as a drug against neoplasms [21].

#### 4.1.3 Mode of action

Although several bioactive compounds in MO have anti-cancer properties, studies have uncovered that isothiocyanates play a particularly important role in manipulating the carcinogenic pathway in skin cancer. Wang et al., 2019 prove that 12-*O*-tetradecanoyl-phorbol-13-acetate (TPA)-carcinogenic changes including deviations in nuclear factor erythroid 2-related factor (Nrf2)-regulated redox pathways, nuclear factor kappa-light-chain-enhancer of activated B cells (NF-kB), Interleukin-1 (IL-1), retinoid X receptor (RXR), phosphatase and tensin homolog deleted on chromosome 10 (PTEN) and p53 pathways in cancerous epidermal cells of a JB6 mouse were able to be reversed using MO [22].

**Fig. 1:**
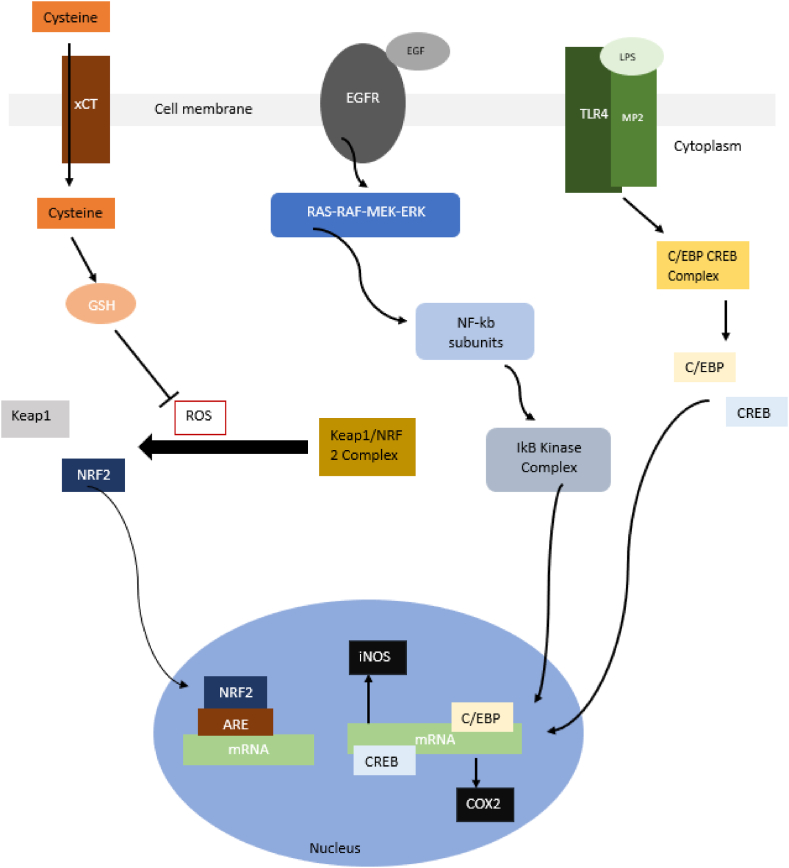
A simple and brief depiction of Pathways 1–4.

As seen in Fig. 1, MO can alter the activity of several pathways to prevent carcinogenic activity. The main pathways manipulated are [22].

##### 4.1.3.1 Pathway 1

a) When exposed to oxidative conditions, the Keap1/NRF2 complex within the cytoplasm dissociates into Keap1 and NRF2.
b) NRF2 enters the nucleus, binds onto mRNA and nuclear translocation follows.
c) The translocation process is then finalised with the help of coactivators and corepressors.
d) The transcribing phase of detoxifying genes that follows is induced only in the presence of the antioxidant response element (ARE) which has to be bound to the mRNA and NRF2.

##### 4.1.3.2 Pathway 2

a) Glutathione (GSH) is activated via the intracellular secretion of cysteine which is expelled via the cysteine/glutamate antiporter (xCT).
b) This in turn promotes the trapping of reactive oxygen species (ROS). This free radical scavenging leads to the inhibition of step a in Pathway 1.
c) Therefore, the KEAP1/NRF2 complex does not dissociate.

##### 4.1.3.3 Pathway 3

a) Concurrently, the intracellular RAS-RAF-MEK-ERK pathway is triggered by the communication between the epidermal growth factor (EGF) and its ligand epidermal growth factor receptor (EGFR).
b) This results in NF-kB subunits – p50 and p65-to be secreted.
c) The IkB Kinase complex then stimulates the expelled subunits to be translocated on the nucleus.
d) Inducible nitric oxide synthase (iNOS) transcription is also prompted.

##### 4.1.3.4 Pathway 4

Simultaneously, induction of lipopolysaccharide (LPS)-stimulated Toll-like receptor-Macrophage 2 (TLR4-MP2) encourages cyclooxygenase-2 (COX-2) transcription. This results in nuclear translocation of the CRED-C/EBP complex.

##### 4.1.3.5 Other pathways

Janus kinase-signal transducer and activator of transcription pathway (JAK-STAT5), phosphoinositide 3-kinase (PI3 Kinase) and protein kinase B (PKB) pathways are manipulated analogy by MO too [22].

#### 4.1.4 Supporting evidence of *Moringa oleifera* as an anti-carcinogenic

Many experiments involving animal tissue have been carried out to better understand the actions of MO in cancer. MO was able to reduce cell proliferation via its cytotoxic and ribosomal ribonucleic acid (rRNA) degradation properties in the human A549 lung cancer cell line. In fact, normal non-cancerous cells were unaffected by the extract indicating the possible protective property of the extract in healthy cells against severe cytotoxicity [18]. Furthermore, anti-carcinogenic activity in human colorectal adenocarcinoma (HT-29), human colorectal carcinoma (HCT-116), oral squamous cell carcinoma (ORL-48) and human leukemic cell lines have also been reported [19]. Similarly, root and leaf extracts obtained from the plant can help fight prostate, pancreatic, breast and ovarian cancers as well as protect against the advancement of ulcerative colitis to colorectal cancer [6,22].

In conjunction with the above, in a dose-dependent manner, MO can bring about a change in mitochondrial membrane potential through the secretion of cytochrome *c* which in turn initiates a cascade of reactions that will result in apoptosis. When tumour-bearing mice were treated with an aqueous extract of MO, this feature together with tissue architecture restoration was not only seen to reduce tumour growth but it prolonged the survival rate and lifespan of the mice by a two-to-four-fold [19].

### 4.2 *Moringa oleifera* and the cardiovascular system

Several studies conducted primarily on different rat species have shown the cardioprotective effects of MO. The main phytochemicals responsible for the antifibrotic, antihypertensive and hypolipidemic properties contributing to these characteristics are niazirin, niaziridin, niazinin A, niazinin B, niazimicin, β-sitosterol, nitriles, flavanoids and thiocarbonates glycosides [1,23].

#### 4.2.1 Cardioprotective effects of *Moringa oleifera* in spontaneous hypertensive rats

Treating spontaneous hypertensive rats with contents attained from MO seeds has depicted improvements in cardiac diastolic function including cardiac output and ejection fraction as well as a reduction in nocturnal heart rate and epsilon wave duration on an electrocardiogram (ECG) without any alteration in blood pressure [23]. Furthermore, remodelling of the cardiac tissue promoted a diminution in the anterior left ventricular and interseptal walls’ thickness which was unfortunately altered by the cardiopathy [13].

In addition, as depicted by Randriamboavonjy et al. (2016) and Rani et al. (2018) MO could amplify proliferator-activated receptor-α (PPAR-α) and PPAR-δ nuclear receptors' expression. This in turn increased their activities as well as the levels of plasmatic prostacyclin. This revelation brought to light the decrease in cardiac triglyceride levels via beta fatty acid oxidation in the rats’ hearts which alleviated their risk of developing cardiovascular associated diseases like atherosclerosis and hyperlipidaemia.

It is also believed that MO exerts its effects by manipulating signalling pathways specifically the calmodulin-activated serine-threonine protein phosphatase calcineurin pathway since it was able to inhibit the activity of cardiac calcineurin which increases with age. By doing so, it prevents the development and/or severity of hypertension [23]. Therefore, in this case, through the modification of the pathway involved in pressure overload-induced left ventricular hypertrophy through a calcium handling mechanism, MO's cardioprotective and anti-hypertensive role is depicted [13,23].

#### *4.2.2* *Moringa oleifera* in isoproterenol induced myocardial infarction in Wistar rats

Another study involving Wistar rats which were induced with myocardial infarction using isoproterenol specifically observed the cardioprotective role of methanolic extract of *Moringa oleifera* seeds (MEMOS) through alterations in myocardial marker enzymes - lactate dehydrogenase, creatine phosphokinase, aspartate transaminase, alanine transaminase, creatine phosphokinase-MB and lipid profiles - total cholesterol (TC), triglyceride (TG) [24].

Unfortunately, isoproterenol is known to increase serum myocardial marker enzymes whilst decreasing cardiac myocardial marker enzymes simultaneously. Providentially, MEMOS can return both the cardiac and serum levels of the myocardial marker enzymes close to normality. Similarly, the high lipid profiles in the diseased rats were also assuaged to healthier parameters using MEMOS. Interestingly, a decrease in TC, TG and low-density lipoprotein and an increase in high-density lipoprotein levels were noted in the rats that were pre-exposed to MEMOS [24]. It is suggested that these cardioprotective characteristics are acquired through MEMOS’ antioxidant properties that can attenuate lipid peroxidation and lipid accumulation, maintain ideal glutathione levels and reduce cardiac inflammation [6,24]. Other *Moringa oleifera* extracts that have cardioprotective roles.

MO's root bark is rich in moringinine. Moringinine can influence the heart rate by regulating the cholinergic and sympathetic systems which have a vital role in cardiac performance [1,23]. Continually, ethanol extract obtained from the plant's leaves has proven its ability to reduce pulmonary arterial blood pressure in induced monocrotaline rats by scavenging free radicals and enhancing glutathione activity [13]. Furthermore, whilst different products produced from the tree, like juice or tea can control and prevent hypertension, the tree's fruit is known to be the most effective in preventing cardiovascular as well as other systemic diseases like fatty liver [11]. Therefore, with respect to all of the above, MO should be considered as a potential therapeutic agent for managing and treating cardiovascular disease in the future.

### *4.3* *Moringa oleifera*, hyperglycaemia and diabetes

The phytochemicals cryptocholinergic acid, quercetin-3-glucoside, kaempferol-3-*O*-glucoside, glucomoringin, phenols, fibre, MO leaf protein isolate and flavonoids in MO exhibit a potent effect in maintaining homeostatic blood glucose levels [6,17]. Therefore, studies on rats and humans have been and are still being conducted to better understand the underlying mechanisms of how MO exerts its anti-hyperglycaemic effect. This will provide new opportunities for potential MO-based drugs that can be used in the management of diabetes mellitus (DM), especially since MO is an innocuous substance displaying no adversative reactions [17,25].

#### 4.3.1 Supporting studies of *Moringa oleifera*'s potential as an anti-diabetic

##### 4.3.1.1 In animals

Feeding obese female Wistar rats 600 mg kg^−1^ day^−1^ of ethanolic MO leave extract over 12 weeks resulted in enhanced insulin resistance and regularised blood glucose [17]. In addition, streptozotocin-induced diabetic rats showed an improvement in health when given MO seed powder since it reduced diabetic nephropathy and restored pancreatic and kidney tissue architecture. In an analogous study, when these rats were treated with MO ethyl acetate leave extract for a month, hyperglycaemia and glycosylated haemoglobin (HbA1c) levels decreased [17,21].

Similarly, diabetic rats, experienced normoglycemia upon introducing 25 mg/kg of MO leaves to their diet. Whilst the results varied with time and dose, this particular attribute was due to the phytochemicals, 4-hydroxyphenylacetonitrite and fluoropyrazine, which were isolated in the leaves [17]. Comparably, in obese Zucker rats, the bioactive chemical isotrifolin in MO leaves exhibited anti-dyslipidaemic, anti-hypertensive and anti-diabetic effects [12].

##### 4.3.1.2 In humans

A study concerning menopausal women between the ages of 45–60 years, revealed a reduction of blood glucose concentration by 13.5% when they consumed 7g of MO leaf powder daily for 90 days [17]. Alternatively, type 2 diabetics between 30 and 60 years of age, depicted a 26% and 28% drop in postprandial and fasting glucose levels respectively, when taking 8g of MO leaves for 80 days [17]. Furthermore, a particular study investigated the metabolic effects of MO tea in 15 healthy individuals in their twenties. In one scenario, the individuals were given 200 ml of MO tea before consuming a 200 ml glucose solution whilst in a separate investigation under the same conditions, 400 ml of the tea was administered instead. Their postprandial blood glucose decreased by 17% and 19% respectively [17]. Interestingly, cold tea is ideally drunk to promote as much metabolic activity as possible because whilst cold MO tea has low levels of protein, adequate concentrations of glucomoringin are present which can be converted to moringin by myorinase. Contrastingly, in hot MO tea, myorinase activity is significantly limited since high temperatures denature the enzyme. Therefore, in this case, the enzymatic conversion of glucomoringin into moringin would solely rely on the individual's gut microbiome [26]. Similarly to the above, when cookies are supplemented with MO, hyperglycaemia is reduced within 30–45 min of ingestion and satiety is achieved quicker and maintained for longer periods [17,26].

#### 4.3.2 Mechanisms

**Fig. 2:**
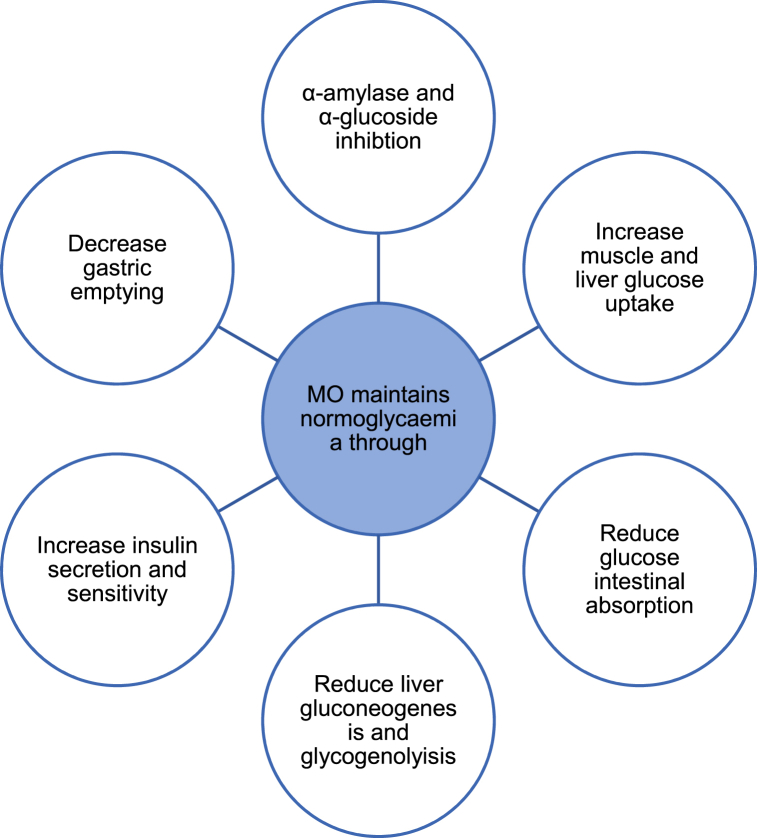
The probable actions through which MO leaves help establish normoglycaemia adapted from Refs. [17,25].

As seen in Fig. 2 MO brings about several changes in one's metabolism to exert its anti-diabetic and anti-hyperglycaemic characteristics. By decreasing gastric emptying and inhibiting the enzymes α-amylase and α-glycosidase which play a vital role in carbohydrate intestinal digestion together with glucose-6-phosphate translocase inhibition which results in reduced hepatic gluconeogenesis and glycogenolysis, MO helps maintain homeostatic levels of blood glucose [12,17].

Furthermore, evidence has shown MO's ability to increase insulin release and sensitivity mainly through the actions of ascorbic acid, fluoropyrazine, methyl-4-hydroxybenzoate, vanillin and 4- α-L-rhamnopyranosyloxybenzyl isothiocyanate. This was achieved by inducing the insulin-dependent protein kinase-A pathway and increasing the expression of the glucose transporter GLUT4 in muscles. This, in turn, promotes the uptake of glucose in the muscles and liver [11,17]. Therefore, MO leaves manipulate several metabolic pathways involved in glucose metabolism to increase insulin sensitivity and secretion, increase glucose uptake by the muscles and liver, reduce the expression of certain enzymes that would increase plasma glucose and reduce glucose intestinal uptake. This aids in achieving homeostatic blood glucose concentrations in diabetics.

It is known that chlorogenic acid isolated in MO promotes proper glucose metabolism. Additionally, studies using rats have revealed that MO leaf extract not only enhances glycogen synthase activity but it also inhibits the formation of advanced glycation end products by reducing monosaccharide glycation [12,17]. Comparably, MO methanol extract acquired from the powder of MO's fruit which is rich in N-benzyl thiocarbamates, benzyl nitriles and N-benzyl carbamates can trigger insulin secretion from the β-cells by downregulating caspase 3 (CASP3) expression which is responsible for causing β-cell apoptosis [21,27]. Through cyclooxygenase suppression and proto-oncogene tyrosine-protein kinase (SRC) and tyrosine-protein phosphatase non-receptor type 1 (PTPN1) modification, insulin release and sensitivity are not only improved but lipid peroxidation is also reduced promoting the maintenance of a healthier lipid profile [27,28].

Moreover, the anti-hyperglycaemic effect of MO's aqueous extract is believed to occur via non-competitive inhibition of glucose transports in the small intestines which leads to a reduction in glucose intestinal absorption. This is particularly favoured by the rich flavonoid glycoside content present in the extract [17,26]. Interestingly, in healthy individuals, a more potent effect of MO's anti-hyperglycaemic activity was recorded at low doses with respect to intestinal glucose absorption whilst a high dose led to a greater influence on circulating glucose [26]. Thus, although more knowledge is required with regards to the pharmacokinetics and pharmacodynamics of MO as an anti-hyperglycaemic, MO does show promising potential as a future therapeutic agent for DM, especially in developing countries where modern drugs are not as accessible [26].

### *4.4* *Moringa oleifera* and obesity

The anti-diabetic and anti-obesity properties of MO go hand-in-hand since MO combats obesity by inhibiting pancreatic lipase and ghrelin, preventing the accumulation of white adipose tissue and maintaining a healthy plasma lipid profile [29]. MO also promotes weight loss through the depression of mRNA leptin and resistin expression whilst increasing the induction of adiponectin gene expression as observed in a study with obese rates [6]. Furthermore, MO enhanced the expression of melanocortin-4-receptor (MC4R) and peroxisome proliferator-activated receptor alpha (PPAR-α) which not only decreases fat aggregation but encourages faster β-oxidation of fatty acids. The plant also protects against the formation of atherosclerotic plaque and restores both liver enzymes and adipokine levels. This strengthens MO's potential as a phytotherapy drug which is similar to simvastatin [29].

### *4.5* *Moringa oleifera*and the nervous system

The Miracle Tree is known to help alleviate the symptoms of neurodegenerative and neuroinflammatory diseases like Alzheimer's Disease (AD) and Parkinson's Disease (PD) [30]. It can also act as an anti-depressant, anti-anxiety agent and protect against cerebral ischemia. This neuroprotective property of MO is linked to its anti-inflammatory and antioxidant characteristics. Additionally, as seen in Table 2 epigallocatechin, gallic acid, palmitic acid, luteolin, oleic acid, salicylic acid alkaloids, saponins, tannins, steroids, glucosinolates and flavonoids are all bioactive compounds present within MO which exert a range of neuroprotective properties. Their common pathway is mainly by preventing the formation of ROS, inflammation and enhancing cognition and memory. This explains why MO can be a potential medicinal plant that can be used to treat anxiety, migraine, dementia and PD [31].

**Table 2:** A depiction of the neuroprotective characteristics of certain phytochemicals found in the MO plant [31].

Phytochemical in MO	Neuroprotective Effect
Alkaloids	Improves the pathophysiology of neurodegenerative diseases through their anti-amyloid, anti-inflammatory, antioxidant, anti-depressant and anti-convulsant characteristics as well as via changes in neurotransmitter systems.
Saponins	Alters neurotransmitter systems together with neurotrophic factors, reduces apoptosis and calcium overload, decreases inflammation and inhibits tau phosphorylation. Neural network regeneration is also enhanced.
Tannins	Prevents neurotoxicity and neural damage caused by oxidative stress by modulating antioxidant enzyme expression.
Steroids	Exerts its neuroprotective role by functioning in blood vessels, neurons and glial cells.
Polyphenols	Has strong antioxidant and free radical scavenging properties.
Glucosinolates	Regulates antioxidant enzyme expression through nuclear factor erythroid 2 – related factor 2/antioxidant response element (Nrf2/ARE) signalling which is induced by protein kinase C- alpha (PKCα) and phosphatidylinositol 3-kinase/protein kinase B (PI3K/AKT) signalling. This improves immunity and reduced oxidative stress.
Flavonoids	Prevents neural damage by neurotoxins, free radicals and inflammations. Memory and cognition are also improved.

#### *4.5.1* *Moringa oleifera* and Alzheimer's disease

Phenols and flavonoids found within methanolic, ethanolic and aqueous extracts of MO have proven their ability to enhance spatial learning, memory, behaviour and motor control. This was seen in a study where a dose of 2 g/kg of MO was administered for a period of time [31]. Furthermore, MO relieves the symptoms of AD mainly through its capacity in preventing neurodegeneration. By enhancing glutathione, catalase, superoxide dismutase, choline acetyltransferase and acetylcholine levels as well as suppressing lipid peroxidation activity and oxidative stress, motor function and cognition are improved [31].

Additionally, MO has also been noted to repair and reduce the extent of injured neuronal networks since it has proline and alanine which are protease inhibitors. Protease inhibitors are capable of reducing the amount of degeneration that occurs in neural diseases [30]. Moreover, the heptanol, *trans*-linaloloxide and linalool oxide isolated in MO extract were observed to cross the blood-brain barrier (BBB) which resulted in the amplification of their therapeutic potential in treating neuronal disorders like AD which involve dysfunction of the BBB [30,31]. In summary, extracts obtained from the plant such as methanolic leaf extract, aid in delaying the progression of the neural disorder as well as managing AD by decreasing behavioural discrepancies, tau hyperphosphorylation, amyloid beta accumulation and oxidative stress which in turn enhances synaptic protein levels, cholinergic activity and memory [32].

#### *4.5.2* *Moringa oleifera*and Parkinson's disease

**Fig. 3:**
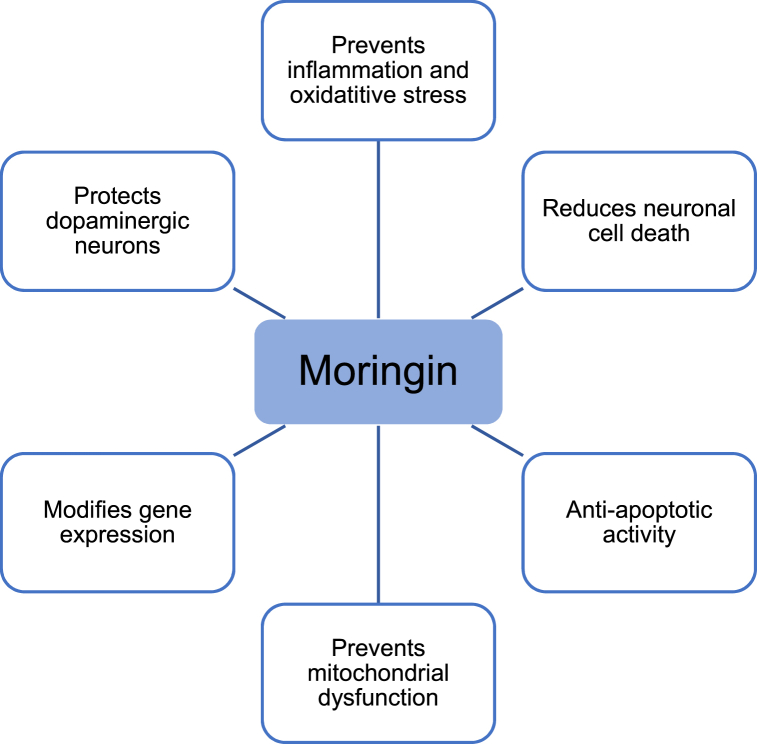
The properties morinigin exerts in combating PD adatped from Ref. [31].

The basic pathophysiology of PD involves inflammation, oxidative stress, protein and mitochondrial dysfunction, autophagy and apoptosis. Luckily, moringin is the main phytochemical in MO that is responsible for relieving the symptoms of patients with PD as seen in Fig. 3. This is seen through its capacity to prevent inflammation, apoptosis, neuronal and mitochondrial dysfunction but most importantly by warding off neuronal cell death. Moringin is released from MO when glucosinolates are hydrolysed [31].

All the characteristics of moringin depicted in the diagram are linked to improving the decline in motor impairments and bradykinesia seen in such patients. This in turn improves their overall behavioural function [31]. In addition, moringin depresses the activity of several pro-apoptotic components including numerous caspases (CASP1, CASP4, CASP6 and CASP8), the superoxide dismutase 1 gene (SOD1) and BCL2-associated X protein (BAX) whilst increasing the activity of the B-Cell lymphoma-2 gene (BCL2) and the myeloid cell leukaemia −1 gene (MCL1) which are anti-apoptotic contributors. In this manner, mitochondria, dopaminergic neurons and neuronal cells are protected from inflammatory and oxidative-induced stress [32]. Dopaminergic neurons are also shielded from injury by moringin's ability to restore tyrosine hydroxylase levels and through the decrease of proinflammatory cytokines and iNOS levels [32].

#### *4.5.3* *Moringa oleifera*, cerebral ischemia and neural development

MO is also cerebroprotective since it can prevent cytotoxicity and oxidative stress not only by its anti-inflammatory and antioxidant properties but also due to its role in reducing ROS production, eliminating intracellular quinone products and preventing membrane and DNA fragmentation. This helps reduce one's risk of brain injury and cerebral ischemia [30]. In fact, studies revealed that in a dose-dependent manner MO can inhibit the development and progression of acute ischemic stroke as MO can decrease neuronal loss, maldistribution and vacuolation of neuronal cell bodies, shrinkage of cell nuclei and necrosis of brain tissue and neurons [31]. Moreover, MO promotes better motor performance and cholinergic activity whilst decreasing brain infarction volume in the cortex and subcortex [31,32].

In addition, ethanolic MO extract has been proven to enhance the branching, number, viability and length of both neurites and dendrites. Apart, from increasing neuronal differentiation, axonal development and synaptogenesis which is mainly due to concentrated β-carotene content, MO reduces cellular injury [30].

#### 4.5.4 Other neuroprotective roles of *Moringa oleifera*

In conjunction with the above, MO extracts can also be used to treat migraines, anxiety and depression. It can also alleviate the symptoms seen in patients having multiple sclerosis and delay the progression of amyotrophic lateral sclerosis (ALS). Furthermore, evidence shows that MO can also be used in the management of epilepsies, in the prevention of convulsions and finally as an analgesic due to its anti-nociceptive properties [1,13,21,30].

### *4.6* *Moringa oleifera* and the reproductive system

#### 4.6.1 In males

In male Wistar rats, an increase in follicle-stimulating hormone (FSH), cholesterol, androgenic and antiperoxidative activity was noted when treated with MO. This study also showed how MO was able to relieve sexual dysfunction via the inhibition of cleaving enzymes monoamine oxidase type B and phosphodiesterase (PDE-5) in male Wistar rats [33]. Similarly, in cadmium-chloride exposed rats, serum testosterone and testicular malondialdehyde improved on exposure to MO. Evidence also supports that MO depresses the production of ROS, preserves the mitochondrial membrane, DNA and acrosome integrity whilst also improving sperm motility, morphology, histological architecture, spermatogenic maturation and spermatogenesis. These fertility-enhancing attributes are due to MO's anti-oxidative phytoconstituents [33]. Contrarily, in an alternate study involving male Wistar rats, upon ingesting the seeds, flowers or roots of MO, the rats depicted contradictory results with respect to fertility. However, other studies have also proven the plant's ability to reverse testicular histopathologies in rats and alleviate hyperprolactinemia-induced male infertility in a dose-dependent manner [33]. Similarly, in a human-conducted study using aqueous MO leaf extract, overproduction of ROS in the sperm was repressed and premature capacitation and acrosome reaction together with nuclear DNA strand breakage was prevented. Likewise, sperm motility, vitality and viability were also improved [33].

#### 4.6.2 In females

Aqueous and alcoholic extracts obtained from the root or stem bark of the Miracle Tree have depicted antifertility properties in postcoital rats and have resulted in foetal resorption during pregnancy. MO acted as an anti-progestational agent that depressed decidual development and the necessary protein concentration required for proper uterine development in female rats [13]. In addition, the plant's oestrogenic and oxytocic properties, enhanced uterine contractions preventing successful implantation, impacting ovum transport negatively, over-proliferation of the vaginal epithelium creating an undesirable environment for pregnancy and impairing proper ova expulsion [34]. Evidence suggests that giving a dose of 100 mg/kg of the extract can have the highest rate of abortion, even up to a 100%. However, despite the abortive effect of MO due to its high phenolic, phytosteroid, alkaloid and saponin content, it has depicted no teratogenic properties so far since the litters birthed from the female rats treated with MO were healthy without any defects [34]. This suggests that while MO has the potential to treat infertility in males, it may also be used to develop a plant-based contraceptive for females [33,34].

### *4.7* *Moringa oleifera*as an anti-microbial

#### *4.7.1* *Moringa oleifera*'s anti-viral properties

Studies have shown that aqueous leaf extract of MO exhibit a strong and specific inhibitory activity on the human immunodeficiency virus (HIV), limiting the virus’ infectivity. This attribute is especially observed when methanol extract is administered as viral infectivity is impeded by 50%. In fact, in a dose-dependent manner, MO is capable of inhibiting the early stages of the viral replication cycle on HeLa cells [16]. Furthermore, MO *p*-miRs have a vital role in regulating the pathophysiology of HIV as it can selectively stimulate the apoptosis and abolition of HIV-infected central memory (CM) CD4 T cells which prevent lymphocyte induction [35]. Such activity was only recorded in HIV positive patients that had a CD4 T cell count greater than 200/mmc. Furthermore, the expression of BCL2, viral DNA integration and intracellular HIV p24 protein were notably decreased following the introduction of MO [35]. Apart from the antiretroviral activity exhibited by MO, the bioactive compounds – flavonoids, saponins and tannins-contribute to reducing viral infection, viral entry, reverse transcriptase and viral transcription activity of HIV *trans*-activator of transcription (tat) and inhibits protease activity [16].

Moreover, MO can be used as a remedy for herpes simplex virus type 1 (HSV-1) as it stops plaque formation by over 50% and delays the development of the virus preventing the formation of skin lesions. In fact, in a study using HSV-1 infected mice, such treatment was able to prolong their lives. MO is also effective in managing the acyclovir-resistant variant of HSV-1 ^13^. Furthermore, this plant also offers protection against poliovirus, respiratory syncytial virus and Epstein-Barr virus [1,16].

#### 4.7.2 Anti-bacterial, anti-fungal and parasitic properties of *Moringa oleifera*

Flavonoids, tannins, steroids, alkaloids, saponins, benzylglucosinolate and benzyl isothiocyanate present in MO exert powerful antimicrobial activity [6]. Additionally, water-soluble lectin which is isolated from MO seed extract depicts inhibitory activity on the growth, cell permeability and survival of several pathogenic species [21]. Although MO can combat both gram-positive (*Staphylococcus aureus* and *Enterococcus faecalis)* and gram-negative bacteria (*Escherichia coli, Salmonella* and *Pseudomonas aeruginosa*), it is more potent in treating gram-positive infections [3,21].

Both methanolic and ethanolic MO extracts impede the growth of oral pathogens reducing the development of dental caries and periodontal diseases. This has led to the incorporation of MO in mouthwash and toothpaste [2,13]. Moreover, MO is also known to aid in the treatment of urinary tract infections, sore throats and pathogenic infections caused by mycobacterium, *Trypanosoma brucei* and waterborne microbes like *Vibrio cholera* [1,6,11,13].

The roots and flowers of MO contain the bioactive chemical, pterygospermin, which is efficient in killing not only bacteria but also pathogenic fungi [11]. Furthermore, the leaves, roots, stems and flowers of MO also exhibit anti-leishmanial activity by inhibiting the parasite's viability and activity in the spleen and liver. Interestingly, MO exerts its anti-microbial activity best at low concentrations [13]. Moreover, apart from MO's immunity-boosting properties, the ethanolic leaf extract is capable of killing the Indian earthworm *Pheritima posthuman* as well as tapeworms [1].

### *4.8* *Moringa oleifera*, the liver and the gut

Flavonoids, ascorbic acid, quercetin, biphenols, tannins and alkaloids exert hepatoprotective effects in preventing and reducing liver fibrosis and damage through their anti-inflammatory properties [6,11]. MO protects the liver's tissue from harmful insults that can disrupt tissue architecture. In fact, ben oil preserves the structural integrity of the hepatocellular membranes and suppresses any liver damage caused by carbon tetrachloride (CCl_4_). Additionally, in hepatic injury, MO prevents the increase in serum aspartate aminotransferase and alanine aminotransferase levels whilst also protecting the liver from drug toxicity that may be induced by rifampicin, isoniazid or paracetamol [3,12].

Furthermore, the phytoconstituents in the methanolic leaf extract of MO, have also exhibited anti-ulcer and anti-gastric lesion qualities in numerous dose-dependent studies. MO acts by depressing gastric volume, the levels of pepsin secreted and the free and total acidity present [3,36]. The cytoprotective property exerted through β-sitosterol also reduces acidity in the pylorus and the risk of developing ulcers whilst also enhancing faster healing of gastric lesions [1,36]. Interestingly, doses of 200 mg/kg of MO showed a protective index against gastric ulcer development of 74% which is very close to the protective index of the commonly used drug, omeprazole, which is 77% [36].

On a similar note, aqueous extract from the seeds of MO depicted an inhibitory activity against acetylcholine-induced contractions in the gut indicating the plant's antispasmodic property which aids the relaxation of intestinal muscles preventing diarrhoea and protecting against dysenteric infections [11,13].

### 4.9 Other pharmacotherapeutic properties associated with *Moringa oleifera*

As evidently depicted MO's medicinal attributes are greatly due to the plant's antioxidant and anti-inflammatory properties. Ben oil extracted from the seeds of the plant can relieve pain and swelling in chronic inflammatory diseases like diabetes, cancer, sepsis, colitis, arthritis, rheumatism and gout by interacting with the inflammatory pathway. Nitric oxide and prostaglandin production can be enhanced by the inflammatory cytokines, interleukin-1 beta (IL-1β) and tumour necrosis factor-alpha (TNF-α). This results in the induction or enhancement of iNOS, COX-2 and microsomal prostaglandin E2 synthase-1 (mPGES-1) activity in specific target cells. As a result of lipopolysaccharide – stimulated human monocyte-derived macrophages, MO is also capable of depressing TNF-α, interleukin- 6 and interleukin- 8 levels whilst simultaneously suppressing the expression of NFkB p65 signalling during inflammation. This is how MO regulates and prevents excessive inflammation [21]. Due to these attributes, MO can act as an anti-asthmatic too since it prevents instantaneous hypersensitivity reactions by reducing interleukin levels in conjunction with its bronchodilator effect. Similarly, ethanolic MO seed extract suppresses cutaneous anaphylaxis induced by anti-immunoglobulin G and histamine secreted by mast cells. This is achieved through its membrane-stabilisation potential. In this way, MO protects against adverse allergic reactions [6].

Furthermore, MO has been reported to improve mean corpuscular haemoglobin and platelet concentrations in anaemics, improve thyroid function, alleviate lower back pain, reduce fever and protect against retinal dysfunction induced by diabetes as well as the development of cataracts and blindness [1,6,11]. Moreover, MO leaf, seed and dried pulp extracts were observed to ameliorate hydroxyproline content, endothelial growth factor activity, wound closure and healing rate and granuloma-breaking strength as well as depressing scar area in rats. This depicts MO's wound resolution abilities [1,6]. In addition to MO's capacity to cure and/or treat over 300 diseases, MO can act as a diuretic and anti-urolithiatic too since it prevents the retention of calcium, oxalate and phosphate in the kidneys [1,12]. It can also be used in the cosmetics industry as skin moisturisers, shampoos, conditioners, deodorants, perfumes and sunscreen [8].

## 5 *Moringa oleifera* and the coronavirus disease 2019 (COVID-19)

Since the first case of the coronavirus disease 2019 (COVID-19) on December 31, 2019 and the declaration of this disease as a global pandemic on March 11, 2020, the race to find an effective drug to treat COVID-19 had begun. Unfortunately, till now March 2022, no effective and standard treatment has been found [37]. Due to the high infectivity and mortality rate of this disease, caused by the virus Severe Acute Respiratory Syndrome Coronavirus-2 (SARS-CoV-2), together with its resistance to various drugs and high mutation rate, the urgency for a therapeutic agent to combat this virus has surged [37,38]. Several plants have been used in Chinese and Ayurvedic medicine for centuries due to their anti-viral and anti-microbial properties. Thus, MO*'*s phytocomposition and numerous attributes have made it an attractive plant to be researched as a possible treatment for COVID-19 ^39^.

Infection with COVID-19 results in severe inflammation due to the cytokine storm effect which can easily result in organ failure. This effect is initially caused by chemical signals induced by neutrophil chemotaxis by chemicals like interleukin-6/8. Upon assembly, these chemicals secrete more inflammatory components which leads to a cascade of reactions amplifying the inflammatory response. Unfortunately, the non-structural proteins 9 and 10 (nsp9, nsp10) present in COVID-19, promotes this inflammatory response [40]. In a study using quantum chemical, molecular docking and dynamic methods conducted by Muhammad et al., 2021 it was concluded that the phytochemicals, ellagic acid and apigenin isolated from MO, had the greatest binding affinities of −7.1 kcal/mol and −6.5 kcal/mol against nsp9 and -6.9 kcal/mol and −7.1 kcal/mol against nsp10 respectively. Additionally, these two compounds exhibited a high intestinal absorption and clearance rate due to being water-soluble and were also able to inhibit viral growth. Furthermore, ellagic acid and apigenin showed no interference with the drugs metabolised by cytochrome P2D6 (CYP2D6) and cytochrome P3A4 (CYP3A4) which are isoenzymes of cytochrome P450 (CYP450) as their activity was not altered. They also showed no mutagenic or toxic effects making MO a safe potential phytotherapy for COVID-19 ^40^.

Viral replication in COVID-19 is vastly aided by main protease (M^pro^). This makes M^pro^ an enticing target for anti-COVID-19 drugs [37]. Sen et al., 2021 investigated the interaction between MO and M^pro^. It revealed that the flavonoids isorhamnetin, kaempferol and apigenin had incredible binding affinity and formed stable protein-ligand complexes when binding with M^pro^. This was also seen with the glycosidic forms of the phytochemicals, quercetin-3-rhamnoside, myricetin-3-rutinosie and rutin. Through this interaction, M^pro^ can be suppressed and consequently so can viral replication. Since these phytoconstituent acted similarly to the anti-viral drug baicalein, it further solidifies *Moringa oliefra*'s potential as a preventive and anti-viral agent against COVID-19 ^37^.

Similarly, Mathpal et al., 2021 stated that kaempferol-3-*O*-rutinosde and vitexin found in MO had high binding affinity with M^pro^ and formed stable complexes. In addition, since these compounds were water-soluble, they could easily be absorbed from the intestines and could penetrate the blood-brain barrier. This cemented their aptitude to perform as drugs.

In addition, Nair and J, 2020 analysed the binding affinity of many phytochemicals present in MO with M^pro^ and compared their binding affinities and other criteria like intestinal absorption with anti-viral FDA approved drugs as seen in Table 3. Most of the phytochemicals listed in the table either showed a higher or slightly lower binding affinity with Mpro than the FDA approved anti-viral drugs mentioned. However, unlike the anti-viral FDA approved drugs which were all P-gp substrate and have a low intestinal absorption rate (except for Maraviroc), this was not the case for some of the phytoconstituents. This in-silico study revealed the potential for the development of a MO-based drug that can prevent COVID-19 drug resistance as well as similarly inhibit Mpro as the FDA approved anti-viral drugs [39]. Finally, although apigenin-7-*O*-rutinoside depicted the greatest affinity with Mpro, all the other phytochemicals still showed potential against the virus as well as antioxidant activity which can help improve post-COVID secondary infection.

**Table 3:** An analysis of the pros and cons of the phytochemicals that can inhibit Mpro activity [37,41]. *(X kcal/mol) describes the binding affinity of the drug or phytochemical with Mpro.

Phytochemical in MO	Pros	Cons
Apigenin-7-*O*-rutinoside (−8.8 kcal/mol)*	Higher binding affinity with M^pro^ than the FDA approved antiviral drugs Raltegravir (−7.2 kcal/mol)*, Lopinavir-Ritonavir (−7.7 kcal/mol)*, Maraviroc (−8.2 kcal/mol)* and Nelfinavir (−8.3 kcal/mol)*	P-glycoprotein (P-gp substrate). Low intestinal absorption rate Low bioavailability score
Mudanpioside J (−8.3 kcal/mol)*	Higher binding affinity with M^pro^ than the FDA approved antiviral drugs Raltegravir (−7.2 kcal/mol)*, Lopinavir-Ritonavir (−7.7 kcal/mol)*, Maraviroc (−8.2 kcal/mol)* and showed the same affinity as Nelfinavir (−8.3 kcal/mol)*	Low intestinal absorption rate
Isoquercetin (−8 kcal/mol)* and Hyperoside (−7.8 kcal/mol)*	Higher binding affinity with M^pro^ than the FDA approved antiviral drugs Raltegravir (−7.2 kcal/mol)* and Lopinavir-Ritonavir (−7.7 kcal/mol)*	Low intestinal absorption rate
Quercetin (−7.8 kcal/mol)* and Dihydroquercetin ((-7.8 kcal/mol) *	Higher binding affinity with M^pro^ than the FDA approved antiviral drugs Raltegravir (−7.2 kcal/mol)* and Lopinavir-Ritonavir (−7.7 kcal/mol)*	High intestinal absorption rate
Neochlorogenic acid (−7.3 kcal/mo)*	Lower binding affinity with M^pro^ than the FDA approved antiviral drugs Lopinavir-Ritonavir (−7.7 kcal/mol)*, Maraviroc (−8.2 kcal/mol)* and Nelfinavir (−8.3 kcal/mol)*	Low intestinal absorption rate Low bioavailability score
Catechin (−7.5 kcal/mol)* and Epicatechin (−7.4 kcal/mol)*	Higher binding affinity with M^pro^ than the FDA approved antiviral drug Raltegravir (−7.2 kcal/mol)*	High intestinal absorption rate High bioavailability score
Rhamnetin (−7.2 kcal/mo)* and Niazirin (−7 kcal/mol)*	Lower binding affinity with M^pro^ than the FDA approved antiviral drugs Lopinavir-Ritonavir (−7.7 kcal/mol)*, Maraviroc (−8.2 kcal/mol)* and Nelfinavir (−8.3 kcal/mol)*	High intestinal absorption rate High bioavailability score

Furthermore, other studies explored the binding affinities of MO's phytochemicals with SARS-CoV-2 spike glycoproteins and human angiotensin-converting enzyme 2 (ACE2) receptor which revealed positive and promising results as a potential phytotherapy to combat the ongoing pandemic [42].

Since the onset of the pandemic, in 2020 over 49 million people entered a state of poverty and over the past two and a half years, it was projected that over 130 million individuals experienced extreme hunger [43]. This will result in a surge in morbidity and mortality rates due to poor living conditions and weak immune systems. Fortunately, MO is an inexpensive, accessible and easily cultivated tree that can boost one's immune system due to its rich niaziminin B, vitamin A and iron content [38,39]. This can aid in the prevention of COVID-19 infection and transmission.

Apart, from boosting one's immunity, good personal hygiene is needed to prevent virus transmission. Luckily, extracts obtained from MO leaves have the potential to be incorporated into hand sanitisers due to the phytochemical's antiseptic activity as depicted in Table 4 [44]. This is possible since phytochemicals like saponins, triterpenoids, flavonoids, tannins and alkaloids isolated in MO can inhibit the growth of bacteria and other microorganisms. Arifan et al., 2021 stated that using the percolation extraction method, where the raw components spend 24 min in a percolator at a temperature of 40 °C and are stirred for 45 min would yield the best extract quality abiding with hand sanitiser quality standards. This study carries weight as hand sanitisers have become a handy and effective way of maintaining hand hygiene and preventing microbe transmission.

**Table 4:** The antiseptic activity of *Moringa oleifera's* phytoconstituents [44].

Phytochemical in MO	Antiseptic action
Flavonoids	Interact with bacterial DNA resulting in the alteration of the permeability of bacterial cell walls, microsomes and lysosomes.
Saponins	Increase bacterial cell wall leakage by increasing their cell wall's permeability. This results in the inadvertent efflux of intracellular components.
Alkaloids	Results in the lysis of bacterial cells due to the alteration in the composition of the peptidoglycan bacterial cell wall.

## 6 The toxicity, safety and pharmacokinetics of *Moringa oleifera*

Nowadays, people are becoming more conscious of the food they eat and from where it is sourced. With the intention to adopt a healthier lifestyle and a healthy balanced diet, people are turning to more nutritious food. Thus, it is no surprise that the use of herbal medicine is increasing globally. However, despite the assumption that all herbs and plants are safe, one needs to have a basic understanding of the toxicity and adverse effects that medicinal plants may have [45].

A study exploring the toxicity levels of aqueous leaf extract of MO on male Wistar albino mice revealed that although no fatalities were observed at doses of 6400 mg/kg, administering doses above 1600 mg/kg for a long time can lead to dullness and sluggishness. In addition, no significant changes in haematological parameters were observed and no significant weight gain occurred indicating no changes in hormone levels and metabolism [45]. Similarly, Stohs and Hartman, 2015 stated that whilst doses of 1000 mg/kg of MO leaf extract depicted no genotoxic activity in rats, the lethal dose was 1585 mg/kg. Furthermore, methanol root extract of MO was observed to lead to hepatoxicity and nephrotoxicity in 24 guinea pigs in a time-dependent manner rather than a dose-dependent relationship. Since intraperitoneal administration of the extract exhibited ballooning liver degeneration at doses of 3.6 mg/kg, 4.6 mg/kg and 7.0 mg/kg for 3 weeks and mild tubular damage and interstitial inflammation in the kidneys at doses of 4.6 mg/kg over 3 weeks. Contrastingly, human studies in which 8 g/day of MO leaf powder was given for 40 days, revealed no adverse effects, indicating MO to be safe, especially at doses that are widely used [46].

Likewise, the alkaloids spirochin and bencil isothiocyanate isolated in the roots and bark of the plant can be toxic as the former can result in tachycardia at doses of MO of 35 mg/kg body weight and kidney injury at constant doses which are greater than 46 mg/kg [47]. It is believed that the lethal dose in 50% of the population when MO leaves are consumed over a long period is 17.8 g/kg body weight. Overconsumption of MO can result in nerve paralysis, liver failure, alterations in haematological parameters and spleen hypertrophy. When the meals of children were fortified with 20 g of MO daily, 20% of the children experienced mild nausea. Similarly, consuming roasted MO seeds can also be harmful due to the potential presence of mutagens like as 4-(α-lramnopyranosyloxy)-benzyl glucosinolate [48]. Moreover, although MO has been reported to ameliorate thyroid performance in hypothyroidism and can alleviate thyrotoxicity [49,50], since MO leaf extract lowers plasma triiodothyronine (T3) whilst increasing plasma thyroxine (T4) levels, high doses of the MO leaf extract may not be safe. Thus, ideally, low doses are used when managing hyperthyroidism [51].

On a different note, studies have investigated the pharmacokinetics of MO as well as its effect on cytochrome enzymes which are vital for proper drug metabolism. Aqueous and methanolic leaf extract of MO, have shown inhibitory effects on CYP3A4 and CYP2D6. In fact, noteworthy inhibition of CYP3A4 was reported at doses of 0.5 mg/ml for methanolic MO leaf extract and 2.5 mg/ml for aqueous MO leaf extract [4]. Moreover, ethanol MO leaf extract has also depicted inhibitory activity on cytochrome P1A2, cytochrome P2D6, cytochrome P2E1 and CYP3A4 in a dose-dependent relationship. The phytochemicals, omoringone, rutin, lariciresinol-9-*O*-β-d-glucopyranoside and isoquercitrin also exhibited great inhibition on CYP3A4 ^4^.

Furthermore, when the plant is to be used for consumption or medicinal use it is vital to consider the environment in which the tree was grown. This is because the seeds, leaves and bark of MO, are able to absorb and take up heavy metals like zinc, arsenic, lead, copper and chromium from their surroundings. Thus, in polluted areas, the plant can accumulate great concentrations of these heavy metals which can easily be passed to other organisms upon digestion. The consequences can be detrimental to one's health [52]. In saying this, it is evident that whilst this multipurpose tree can aid in the prevention and treatment of multiple diseases as depicted in Table 5, caution is needed when MO is consumed over long periods [45].

**Table 5:** A summary of the multiple health benefits of MO.

The health benefits exerted by MO	Mode of action	Reference
Anti-cancer and chemotherapeutic agent	Manipulates the cancerous pathways involved in tumour development and growth via the Keap1/NRF2 complex, GSH, EGF, EGFR and CREP-C/EBP complex. MO has low toxicity levels, high antioxidant properties and tumours show very little resistance to MO making it a great potential chemotherapeutic agent.	[21,22]
Anti-diabetes and anti-obesity	Maintains homeostatic blood glucose levels by reducing gastric emptying and glucose intestinal absorption whilst increasing insulin secretion and sensitivity. Prevents obesity by decreasing white adipose tissue accumulation, maintaining a healthy lipid profile and inhibiting pancreatic lipase and ghrelin.	[17]
Cardioprotective	Improves cardiac diastolic function, cardiac output and ejection fraction. MO prevents hypertension by altering the calmodulin-activated serine-threonine protein phosphate calcineurin pathway.	[23]
Neuroprotective	Alleviates and improves the symptoms seen in AD and PD. In addition, MO protects against cerebral ischemia and depression. It also improves memory. This neuroprotective role is linked to MO's antioxidant and anti-inflammatory properties which are exhibited by luteolin, oleic acid, palmitic acid and other phytochemicals.	[31]
Reproduction System	In males, MO can aid in combating infertility since it can improve sperm viability, motility and vitality. Contrarily, in females, MO can act as a contraceptive due to its abortive property.	[33,34]
Anti-microbial	MO can be used to treat HIV, HSV-1, fungal and bacterial infections and several other pathogenic infections. This is mainly due to the presence of flavonoids, tannins and sterols in MO.	[3,16]
Anti-COVID-19	MO can serve as a potential anti-viral drug against COVID-19 since it suppresses M^pro^. This results in the inhibition of COVID-19 viral replication. MO also reduces the inflammatory response brought about by COVID-19 infection.	[37,40]

## 7 Future prospective of *Moringa oleifera* in health and pharmaceutical industry

The future prospects of MO in the health and pharmaceutical industry are both promising and multifaceted, reflecting the growing demand for natural and sustainable health solutions on a global scale. Within the pharmaceutical domain, ongoing research into MO's significant anticancer, anti-inflammatory, and antimicrobial properties holds significant potential for the development of novel therapeutic agents. The isolation and synthesis of MO's bioactive compounds may lead to the creation of pharmaceuticals with improved efficacy and reduced side effects, offering new horizons for drug development. Moreover, MO's potential in managing chronic diseases, such as diabetes and cardiovascular conditions, presents a substantial opportunity for enhancing the quality of life for a considerable portion of the population.

In the realm of functional foods and nutraceuticals, MO offers a rich nutritional profile with high levels of vitamins, minerals, and antioxidants, meeting the demand for natural, plant-based dietary solutions. As the world increasingly embraces holistic wellness and preventive healthcare, MO's diverse health benefits align with this approach, addressing specific health concerns while promoting overall well-being. To unlock MO full potential for human health and well-being, continued research into its molecular mechanisms, bioactive compounds, and interactions with biological pathways is imperative. This ongoing exploration holds the promise of revolutionising healthcare and pharmaceuticals, offering natural, sustainable, and effective solutions for a broad spectrum of health concerns.

## 8 Conclusion

In conclusion, this comprehensive review underscores the remarkable potential of MO as a versatile botanical resource with substantial therapeutic and nutritional value. Through an in-depth analysis of its phytochemical constituents, health benefits, mechanisms of action, and pharmacological safety, this study elucidates MO's role as a promising candidate for addressing various health concerns. The synthesis of diverse research findings highlights MO's noteworthy anticancer, antioxidant, anti-diabetic, anti-inflammatory, antimicrobial, and cardioprotective effects, among other health-promoting attributes. The examined literature not only reaffirms MO's historical significance in traditional medicine but also underscores its growing importance in modern medicinal practices. The collective evidence suggests that MO's potential extends beyond its nutritional richness to encompass its potent bioactive compounds that interact with intricate biological pathways. However, further research is imperative to unravel the precise molecular mechanisms underlying MO's diverse effects and to bridge the gap between traditional knowledge and modern scientific understanding. As we contemplate MO's journey from traditional remedy to potential modern medicine, it serves as an exemplar of nature's potential to offer holistic solutions for human well-being. By offering insights into MO's therapeutic potential, this review contributes to advancing research in the realm of natural remedies and encourages further exploration of MO's applications in promoting health and preventing disease. Looking to the future, the continued investigation of MO's molecular mechanisms and its incorporation into pharmaceutical and nutraceutical developments holds the promise of harnessing its full potential for the benefit of human health.

## Funding

This research did not receive any specific grant from funding agencies on the public, commercial, or not-for-profit sectors.

## Ethics declaration

Review and/or approval by an ethics committee as well as informed consent was not required for this study because this literature review only used existing data from published studies and did not involve any direct experimentation/studies on living beings.

## Data availability statement

No data was used for the research described in the article. No data associated in this article has been deposited into a publicly available re‵pository.

## CRediT authorship contribution statement

**Emma Camilleri:** Writing – review & editing, Writing – original draft, Methodology, Conceptualization. **Renald Blundell:** Supervision.

## Declaration of competing interest

The authors declare that they have no known competing financial interests or personal relationships that could have appeared to influence the work reported in this paper.

## References

[1] Varmani Shivani, Garg Meenakshi (2014). Health benefits of moringa oleifera: a miracle tree [Internet]. https://www.researchgate.net/publication/341440986_Health_benefits_of_Moringa_Oleifera_A_miracle_tree.

[2] Sujatha Bk, Patel Poonam (2017). Moringa oleifera – nature's gold [internet]. https://www.researchgate.net/publication/317930958_Moringa_Oleifera_-_Nature%27s_Gold.

[3] Razis A.F.A., Ibrahim M.D., Kntayya S.B. (2014). Health benefits of moringa oleifera. Asian pac J cancer prev [Internet]. https://pubmed.ncbi.nlm.nih.gov/25374169/.

[4] Fantoukh O.I., Albadry M.A., Parveen A., Hawwal M.F., Majrashi T., Ali Z. (2019 Jul 1). Isolation, synthesis, and drug interaction potential of secondary metabolites derived from the leaves of miracle tree (Moringa oleifera) against CYP3A4 and CYP2D6 isozymes. Phytomedicine.

[5] Velázquez-Zavala M., Peón-Escalante I.E., Zepeda-Bautista R., Jiménez-Arellanes M.A. (2016). Moringa (Moringa oleifera Lam.): usos potenciales en la agricultura, industria y medicina. Rev. Chapingo Ser. Hortic..

[6] Bhattacharya A., Tiwari P., Sahu P.K., Kumar S. (2018).

[7] Mahfuz S., Piao X.S. (2019). http://www.mdpi.com/journal/animals.

[8] Velázquez-Zavala M., Peón-Escalante I.E., Zepeda-Bautista R., Jiménez-Arellanes M.A., Velázquez-Zavala M., Peón-Escalante I.E. (2016 May 1). Moringa (Moringa Oleifera Lam.): Potential Uses in Agriculture, Industry and Medic.

[9] Saini R.K., Sivanesan I., Keum Y.-S. (2016 Dec 22). Phytochemicals of Moringa oleifera: a review of their nutritional, therapeutic and industrial significance. 3 Biotech.

[10] García Milla P., Peñalver R., Nieto G., Trombetta D. (2021).

[11] Dhakar R.C., Maurya S.D., Pooniya B.K., Bairwa N., Gupta M. (2011 Dec). Sanwarmal . Moringa: the herbal gold to combat Malnutrition. J Nutr Educ [Internet].

[12] Alegbeleye O.O. (2018 Mar 1). How functional is moringa oleifera? A review of its nutritive, medicinal, and socioeconomic potential. Food Nutr Bull [Internet].

[13] Rani N.Z.A., Husain K., Kumolosasi E. (2018 Feb 16). Moringa genus: a review of phytochemistry and pharmacology. Front. Pharmacol..

[14] Wang F., Bao Y., Zhang C., Zhan L., Khan W., Siddiqua S. (2020). Bioactive components and anti-diabetic properties of Moringa oleifera Lam. Crit Rev Food Sci Nutr [Internet].

[15] Giuberti G., Rocchetti G., Montesano D., Lucini L. (2021 Dec 1). The potential of Moringa oleifera in food formulation: a promising source of functional compounds with health-promoting properties. Curr. Opin. Food Sci..

[16] Nworu C., Okoye E., Ezeifeka G., Esimone C. (2016 Apr 12). Extracts of Moringa oleifera Lam. showing inhibitory activity against early steps in the infectivity of HIV-1 lentiviral particles in a viral vector-based screening. African J Biotechnol [Internet].

[17] Ahmad J., Khan I., Blundell R. (2019 Nov 1). Moringa oleifera and glycemic control: a review of current evidence and possible mechanisms. Phyther Res.

[18] Jung I.L. (2014 Apr 18). Soluble extract from moringa oleifera leaves with a new anticancer activity. PLoS One [Internet].

[19] Barhoi D., Upadhaya P., Nath Barbhuiya S., Giri A., Giri S. (2020). https://www.tandfonline.com/action/journalInformation?journalCode=uacn21.

[20] Kou X., Li B., Olayanju J., Drake J., Chen N. (2018 Mar 12). Nutraceutical or pharmacological potential of moringa oleifera Lam. Nutrients.

[21] Kou X., Li B., Olayanju J.B., Drake J.M., Chen N. (2018). Nutraceutical or pharmacological potential of moringa oleifera Lam. Nutrients [Internet].

[22] Sodvadiya M., Patel H., Mishra A., Nair S. (2020).

[23] Randriamboavonjy J.I., Loirand G., Vaillant N., Lauzier B., Derbré S., Michalet S. (2016). Cardiac protective effects of moringa oleifera seeds in spontaneous hypertensive rats. Am J Hypertens [Internet].

[24] .

[25] Haber S.L., McMahon R.P., Barajas J., Hayes A.R., Hussein H. (2020 Oct 30). Effects of Moringa oleifera in patients with type 2 diabetes. Am J Heal Pharm [Internet].

[26] Nova E., Redondo-Useros N., Martínez-García R.M., Gómez-Martínez S., Díaz-Prieto L.E., Marcos A. (2020). http://www.mdpi.com/journal/nutrients.

[27] Huang Q., Liu R., Liu J., Huang Q., Liu S., Jiang Y. (2020).

[28] Bamagous G.A., Al Ghamdi S.S., Ibrahim I.A.A., Mahfoz A.M., Afify M.A., Alsugoor M.H.M. (2018 Jun 1). Antidiabetic and antioxidant activity of ethyl acetate extract fraction of *Moringa oleifera* leaves in streptozotocin-induced diabetes rats via inhibition of inflammatory mediators. Asian Pac J Trop Biomed [Internet].

[29] Ezzat S.M., El Bishbishy M.H., Aborehab N.M., Salama M.M., Hasheesh A., Motaal A.A. (2020 Apr 6). Upregulation of MC4R and PPAR-α expression mediates the anti-obesity activity of Moringa oleifera Lam. in high-fat diet-induced obesity in rats. J. Ethnopharmacol..

[30] Igado O.O., Olopade J.O. (2017). A review on the possible neuroprotective effects of Moringa oleifera leaf extract. Niger J Physiol Sci [Internet].

[31] Ghimire S., Subedi L., Acharya N., Gaire B.P. (2021).

[32] Khan M.F., Yadav S., Banerjee S. (2021 Dec 14). Review article on effects of moringa on central nervous system. J. Young Pharm..

[33] Tebatso F. (2020).

[34] Zade Varsha, Dabhadkar Dinesh (2014). Antifertility effect of alcoholic extract of moringa oleifera stem bark on estrous cycle and estrogenic activity of female albino rat. Am J Adv Drug Deliv [Internet].

[35] Minutolo A., Potestà M., Roglia V., Cirilli M., Iacovelli F., Cerva C. (2021 Feb 11). Plant microRNAs from moringa oleifera regulate immune response and HIV infection. Front. Pharmacol..

[36] Swati M., Kansara S. (2013). http://jpsbr.com/index_htm_files/3_JPSBR13RS3003.pdf.

[37] Sen D., Bhaumik S., Debnath P., Debnath S. (2021). Potentiality of Moringa oleifera against SARS-CoV-2: identified by a rational computer aided drug design method. J Biomol Struct Dyn [Internet].

[38] Fajri M. (2021 Jul 1). The potential of Moringa oleifera as immune booster against COVID 19. IOP Conf Ser Earth Environ Sci [Internet].

[39] Mathpal S., Sharma P., Joshi T., Joshi T., Pande V., Chandra S. (2021). Screening of potential bio-molecules from Moringa olifera against SARS-CoV-2 main protease using computational approaches. J Biomol Struct Dyn [Internet].

[40] Muhammad S., Hassan S.H., Al-Sehemi A.G., Shakir H.A., Khan M., Irfan M. (2021 Mar). Exploring the new potential antiviral constituents of Moringa oliefera for SARS-COV-2 pathogenesis: an in silico molecular docking and dynamic studies. Chem. Phys. Lett..

[41] Nair A.D., Jt J. (2020).

[42] Siddiqui S., Ahmad R., Alaidarous M., Zia Q., Ahmad Mir S., Alshehri B. (2022). Phytoconstituents from Moringa oleifera fruits target ACE2 and open spike glycoprotein to combat SARS-CoV-2: an integrative phytochemical and computational approach. J. Food Biochem..

[43] Pereira M., Oliveira A.M. (2020). Commentary Poverty and food insecurity may increase as the threat of COVID-19 spreads. Publ. Health Nutr..

[44] Arifan F., Broto R.W., Sapatra E.F., Pujiastuti A. (2021 Jan 1). Utilization of moringa oleifera leaves for making hand sanitizers to prevent the spread of COVID-19 virus. IOP Conf Ser Earth Environ Sci [Internet].

[45] Awodele O., Oreagba I.A., Odoma S., Teixeira da Silva J.A., Osunkalu V.O. (2012 Jan). Toxicological evaluation of the aqueous leaf extract of Moringa oleifera Lam. (Moringaceae). J. Ethnopharmacol..

[46] Stohs S.J., Hartman M.J. (2015).

[47] Trigo C., Castelló M.L., Ortolá M.D., García-Mares F.J., Soriano M.D. (2021). Moringa oleifera: an unknown crop in developed countries with great potential for industry and adapted to climate change. Foods.

[48] Tshabalala T., Ncube B., Madala N.E., Nyakudya T.T., Moyo H.P., Sibanda M. (2019). Scribbling the cat: a case of the “miracle” plant, moringa oleifera. Plants.

[49] Tabassum W., Roshni A., Sinha M.P. (2013). Effects of leaf extracts of moringa oleifra on regulation of hypothyroidism and lipid profile. N Save Nat to Surviv [Internet].

[50] Alam M.A., Quamri M.A., Haider N. (2021). Efficacy and safety of Barg-e-Sahajna (Moringa olifera Lam.) in primary hypothyroidism. Drug Metab Pers Ther [Internet].

[51] Tahiliani P., Kar A. (2000). Role of Moringa oleifera leaf extract in the regulation of thyroid hormone status in adult male and female rats. Pharmacol Res [Internet].

[52] Agboola O.O., Orji D.I., Olatunji O.A., Olowoyo J.O. (2016 Sep 6). Bioaccumulation of heavy metals by moringa oleifera in automobile workshops from three selected local governments area, Ibadan, Nigeria. West African J Appl Ecol [Internet].

